# A Deep Insight into the Sialotranscriptome of the Gulf Coast Tick, *Amblyomma maculatum*

**DOI:** 10.1371/journal.pone.0028525

**Published:** 2011-12-21

**Authors:** Shahid Karim, Parul Singh, José M. C. Ribeiro

**Affiliations:** 1 Department of Biological Sciences, The University of Southern Mississippi, Hattiesburg, Mississippi, United States of America; 2 Vector Biology Section, Laboratory of Malaria and Vector Research, National Institute of Allergy and Infectious Diseases, National Institutes of Health, Rockville, Maryland, United States of America; Universidade Federal do Rio de Janeiro, Brazil

## Abstract

**Background:**

Saliva of blood sucking arthropods contains compounds that antagonize their hosts' hemostasis, which include platelet aggregation, vasoconstriction and blood clotting; saliva of these organisms also has anti-inflammatory and immunomodullatory properties. Perhaps because hosts mount an active immune response against these compounds, the diversity of these compounds is large even among related blood sucking species. Because of these properties, saliva helps blood feeding as well as help the establishment of pathogens that can be transmitted during blood feeding.

**Methodology/Principal Findings:**

We have obtained 1,626,969 reads by pyrosequencing a salivary gland cDNA library from adult females *Amblyomma maculatum* ticks at different times of feeding. Assembly of this data produced 72,441 sequences larger than 149 nucleotides from which 15,914 coding sequences were extracted. Of these, 5,353 had >75% coverage to their best match in the non-redundant database from the National Center for Biotechnology information, allowing for the deposition of 4,850 sequences to GenBank. The annotated data sets are available as hyperlinked spreadsheets. Putative secreted proteins were classified in 133 families, most of which have no known function.

**Conclusions/Significance:**

This data set of proteins constitutes a mining platform for novel pharmacologically active proteins and for uncovering vaccine targets against *A. maculatum* and the diseases they carry.

## Introduction

Saliva of ticks is a complex mixture of pharmacologically active compounds that interact with their host's hemostasis (the combined result of platelet aggregation, vasoconstriction and blood clotting) and inflammatory reactions that might disrupt feeding [1], [2]. In addition to helping ticks to feed by its biological activities, tick saliva can also enhance pathogen transmission, either by co-feeding ticks [3], or by helping the survival of the pathogen in its adaptation to the new vertebrate host [4], [5], [6], [7]. Because of its dual role in feeding and pathogen transmission, anti-tick saliva vaccines have been proposed as both anti-tick and/or anti tick-borne disease vaccine targets [8], [9], [10].

The combined mixture of these salivary compounds, the sialome (from the Greek sialo = saliva), can be partially uncovered by sialotranscriptome studies that are revealing several hundred different proteins in different tick species [2]. These studies also reveal that the salivary proteins of these organisms are at a very fast pace of evolution, probably due to their host's immune pressure, and perhaps for this reason individual genera, or subgenera of such arthropods have several unique protein families ( = no similarity matches to known proteins at the amino acid sequence level) and that many salivary proteins are products of gene duplication [11], thus creating the scenario for divergent evolution among members of these families.

Sialotranscriptomes have been produced, so far, by Sanger sequencing of cDNA libraries. The number of such sequences, also known as expressed sequence tags (EST's) varies per study from 500 to several thousands [12], [13], [14], [15], [16], [17], [18], [19], [20], [21], [22], [23], [24]. However, the upcoming of the “next generation” sequencing in the form of pyrosequencing allows for cheaper sequencing of millions, not thousands, of sequences thus permitting a much deeper insight into rarer transcripts than previously done.

The Gulf Coast tick *Amblyomma maculatum* is found in the American states surrounding the Gulf and in the Eastern Atlantic region. It is a catholic 3-host tick, immatures of which feed on small rodents and ground birds, while the adults feed on large mammals, being an economical pest of cattle [25]. It can produce tick paralysis in humans [26], [27], to transmit *Hepatozoon americanum* to dogs [28], [29], [30] and to harbour *Rickettsia parkeri* in the US [31]. We here report an annotated catalogue of salivary gland expressed transcripts from adult females of *A. maculatum* resulting from over 1.5 million pyrosequencing sequences, representing the deepest analysis of any sialotranscriptome performed so far. This catalogue should represent a knowledge platform for the discovery of novel pharmacologically active proteins, novel vaccine targets and novel immunoepidemiological markers of tick exposure.

## Methods

### Ticks and Salivary Gland (SG) Preparation

Pathogen-free *Am. maculatum* adult ticks were obtained from Oklahoma State University's tick-rearing facility. All unfed ticks were maintained in the laboratory at 23°C and >90% relative humidity under a 14-hour light/10-hour dark photoperiod before infestation on a sheep according to the methods of Patrick and Hair [32]. Adult ticks were fed on sheep in accordance with protocol # 10042001 approved by the Institutional Animal Care and Use Committee at the University of Southern Mississippi specifically for this study. The partially blood-fed (2, 3, 4, 5, 6, 7, & 9 days post attachment) female adult ticks were dissected within four hours after removal from the sheep. Tick SGs were dissected from 20–30 female ticks from each feeding stage. The dissecting solution was ice cold 100 mM 3-(N-Morpholino)-propanesulfonic acid (MOPS) buffer containing 20 mM ethylene glycol bis-(β-aminoethyl ether)-N, N, N′, N′-tetraacetic acid (EGTA), pH 6.8. After removal, glands were washed gently in the same ice-cold buffer. The dissected SGs were stored immediately after dissection in RNAlater (Ambion Inc., Austin, TX, USA) prior to isolating mRNA.

### RNA Preparation

Poly A^+^ mRNA was isolated from tick SGs using the Illustra™ QuickPrep micro mRNA purification kit (GE Healthcare, Piscataway, NJ, USA) following the manufacturer's protocol. The quality of the mRNA samples was confirmed by lab-on-chip analysis using the 2100 Bioanalyzer (Agilent Technologies, Inc., Santa Clara, CA, USA). The mRNA quantity was determined by a Nanodrop, and the mRNA samples (A260/280>1.8) were pooled for further cDNA synthesis.

### Library Preparation and Sequencing

Library preparations for GS FLX titanium (Roche/454 Life Sciences, Branford, CT, USA) sequencing were developed in the Center for Genomics and Bioinformatics, Indiana University, based on methods for use in GS FLX standard sequencing described in [33] with modifications (K. Mochaitis, unpublished). Briefly, cDNA was synthesized from 1.2 µg of pooled mRNA (2–9 days-post feeding) in a manner similar to Clontech SMART protocols, using primers optimized for the 454 sequencing process and amplified by PCR to generate dsDNA. The cDNA library was normalized using Trimmer cDNA normalization kit (Evrogen, Moscow, Russia) according to manufacturer's instructions. Normalized DNA was then fragmented by sonication, and ends were enzymatically blunted and ligated to customized 454 adaptors. Amplification of ligation products exploited adaptor-mediated PCR suppression [33], [34]. This procedure induces homo-mediated fragment hairpins, thereby severely limiting amplification of mis-ligated products. All amplification steps utilized high-fidelity polymerases. Final library was size selected by excision of the 500–800-bp fraction from the agarose gel. This size selection may have reduced the probability of finding small transcripts such as single Kunitz domain proteins and antimicrobial peptides. Emulsion PCR reactions were performed according to the manufacturer (Roche 454 Life Sciences). To optimize the pyrosequencing throughput, the final libraries were titrated by emulsion PCR bead enrichment prior to sequencing. Sequencing of the salivary cDNA library was performed on a picotitre plate according to the manufacturer's instructions, and yielded 560.4 Mb of sequence data in 1,626,969 read with an average of 344 nucleotides (nt) in length. Sequencing adapters (A and B) were automatically removed from the reads using signal processing software (Roche 454 Life Sciences).

### Bioinformatics Tools Used

The blastn tool (performed locally from executables obtained at the NCBI FTP site ftp://ftp.ncbi.nih.gov/blast/executables/) [35] and CAP3 assembler [36] were used for EST clusterization, by a decreasing word size inclusion strategy. A master program sequentially sent each EST to be blasted against all ESTs using an initial word size of 200 (BLASTN switch -W 200) and a maximum limit of 1,000 matches (-v 1000, using tabular output mode -m 8). Matches were marked as collected as they were retrieved from the blastn program, and these matched sequences were not sent for blast-n when their turn arrived, thus avoiding duplicating the BLAST task. All matches were collected into FASTA and qual-formatted files and fed as input to the CAP3 assembler. The CAP3-outputted FASTA of the assembled data was obtained, including quality files that were then the starting point for the next cycle. This second iteration was done with a word size of 134, the output of which was in turn used for the next assembly round, but now using a word size of 90, then 60, then two more rounds of 40 to produce the final assembly shown in Additional file S1. This assembly strategy is easy to parallelize, allowing for large data sets to be clusterized. In the current case, a total of 48 CPUs were used for 24 hours. The final assembly output was piped into a tab-delimited file that was imported into an Excel spreadsheet, which includes for each assembled contig the number of reads and the list of unique names for each read, to facilitate counting the contribution of different libraries for the final assembly. These operations were automated by a program written in Visual Basic (VB) named Megacluster and associated blaster clients.

Segments of the six-frame translations of the contigs starting with a methionine found in the first 300 predicted amino acids, or the predicted protein translation in the case of complete coding sequences, were submitted to the SignalP server [37] to help identify translation products that could be secreted. To obtain insight on the nature of the transcripts, blastx, blastn, or rpsblast searches of the contigs against several databases were performed. These databases were: the non redundant protein database (NR) of the National Center for Biotechnology Information (NCBI); the Swissprot database; the gene ontology(GO) FASTA subset [38]; the tick salivary sequences described in a previous review [2]; custom downloaded databases from GenBank containing mitochondrial and rRNA nucleotide sequences; and the conserved domains database of NCBI [39] containing the KOG [40], PFAM [41], and SMART [42] motifs.

Sequences matching 50% or more of the length of proteins in the NR, Swissprot, or tick salivary databases had their coding sequences (CDS) automatically extracted by another program written in VB by the senior author to compose the Spreadsheet S2. Because pyrosequencing introduces insertion/deletion (indel) errors in the sequences, this program also recognizes frame shifts in the blastx-derived alignments and marks as N (instead of A, T, C, or G) the nucleotides in the region of frame shift and either subtracts or adds one N to correct the alignment. Spreadsheet S2 was compared by blastp and rpsblast.

Deducted protein sequences were also sent to the SignalP server, to the TMHMM server [43] to detect membrane helices, the NetOglyc server to detect possible mucin-type galactosylations [44] and to the ProP server [45] to identify putative furin-processed protein cleavage sites. The protein sequences in Spreadsheet S2 were also clusterized progressively from 25% similarity to 99% similarity over 50% of the length of the larger sequence, thus helping to identify related protein families.

To functionally classify the protein sequences, another VB program was written that took into consideration key words in the BLAST matches of the Swissprot, GO, tick subset, KOG, PFAM, and SMART databases, as well as their e values, plus the results for SignalP, transmembrane domains, and glycosylation (not used in Spreadsheet S1) to produce nearly 30 functional categories, as indicated in the Spreadsheets S1 and S2. The final results presented were in many cases manually corrected.

Phylogenetic analysis and statistical neighbor-joining bootstrap tests of the phylogenies were done with the Mega package [46] after sequence alignment performed by ClustalW [47].

## Results and Discussion

A total of 1,626,969 pyrosequencing reads were assembled into 190,646 contigs, including singletons. A subset of this data containing only sequences larger than 149 nt yielded 72,441 sequences; these are displayed in Spreadsheet S1. This subset of 72,441 contigs contained 1,498,171 reads, or 92% of the totality of reads. Other parameters of the original reads and assemblies are provided in Spreadsheet S1. A program written by JMCR in VB (see Methods) extracted the coding sequences from the assembled data having at least five reads per contig, generating 15,814 CDS, which derived from 1,251,937 reads, or from 77% of the totality of reads (Table 1). Of these, 5,353 had >75% coverage to their best match in the NR database, and 8,785 had e values by blastp ≤1E-15 when compared with the same database. This annotated set is provided in Spreadsheet S2. Four broad categories of expressed genes are observed in Spreadsheet S2 and summarized in Table 1. The putatively secreted (S) category contained 24% of the reads and had on average 85 sequences per CDS; the housekeeping (H) category had 63% of the reads with an average of 100 reads/CDS; while 13% of the reads with an average of 39 reads/CDS were not classifiable, constituting the Unknown (U) group. Sequences deriving from transposable elements (TE) accounted for 316 contigs, with an average of 24 reads/CDS, and representing 0.6% of the reads. TE-related sequences may indicate either the presence of active transposition in the tick or, more likely, the expression of sequences suppressing transposition. Low-level expression of TE sequences has been a relatively common finding in previous sialotranscriptomes.

**Table 1:** Nature and abundance of reads for extracted coding sequences (CDS) from the sialotranscriptome of *Amblyomma maculatum*.

Class	Number of CDS	Number of Reads	Reads/CDS	Percent of Total Reads
**Secreted**	3475	296284	85.3	23.7
**Housekeeping**	7856	787547	100.2	62.9
**Unknown**	4167	160529	38.5	12.8
**Transposable Elements**	316	7577	24.0	0.6
**Total**	15814	1251937		

The following text is a guide to browsing Spreadsheet S2.

### Housekeeping (H) Genes

The 7,857 CDS attributed to H-class genes expressed in the SGs of *Am. maculatum* were further characterized into 23 subgroups according to function (Table S1 and Spreadsheet S2). Normalization of the library plus the sheer number of reads allowed an unusually deep recovery of transcripts not normally found with limited Sanger-based transcriptomes, such as transcription factors. For example, the *Am. maculatum* homolog of the Enhancer-of-yellow-2 transcription factor was assembled with 920 reads, as was the NFAT transcription factor, assembled from 770 reads. Spreadsheet S2 presents a total of 162 putative transcription factor coding sequences. Of importance for salivary function, 395 coding sequences associated with protein export machinery were retrieved. The vacuolar sorting protein VPS28 was assembled from 1,333 reads. Many vesicular transport-associated proteins—as well as SNARE proteins and members of the signal peptidase complex—were identified and annotated. Native immunity components include proteins annotated as Toll-like receptors and thio-ester/complement-like proteins. Detoxification enzymes were also found, such as sulfotransferases that might be associated with dopamine detoxification [48], [49], a main secretagogue for tick SGs [50], [51]. Enzymes dealing with oxidative detoxification are also abundant, some of which, such as selenoproteins, peroxidasins (haem-peroxidases) and superoxide dismutase may actually be secreted and antagonize inflammatory host responses containing superoxide, nitric oxide or peroxinitrite [52], [53]. Enzymes of the cytochrome P450 family also abound in the sialotranscriptome where 65 contigs were assembled, several of which appear as full length proteins, such as Am-35392 and Am-38412.

### Transposable Elements (TE)

Both class I (retrovirus-like elements) and class II (coding solely for a transposase protein, and having inverted terminal repeats) transposon coding sequences were found in the sialotranscriptome of *Am. maculatum.* All class I–derived coding sequences were truncated by having premature stop codons when compared with active transposons. These products probably act as regulators of transposition or represent remnants of previously active transposition events in the tick genome. On the other hand, seemingly complete transposases of class II elements were identified for the PIF, POGO, and TC1 families, suggesting active transposition of these elements in the tick genome or relatively recent activity.

### Possibly Secreted (S) Class of Expressed Genes

A total of 3,475 coding sequences, derived from 296,284 reads, are possibly associated with a bloodfeeding role as secreted salivary proteins (Table 1 and Spreadsheet S2). These include previously known gene families [2] such as diverse classes of protease inhibitors, metalloproteases, immunomodulators, antimicrobial peptides, basic tail, and glycine-rich peptides. Several protein families were discovered to be specific to *Amblyomma* ticks (because they provide no similarities to other known proteins by blastp), many of which are found to date only on *Am. maculatum*. A total of 133 distinct family classes are annotated in Table S1 and Spreadsheet S2. These families are organized in seven general classes, as described below:

### Protease inhibitor-containing domains

A total of 379 CDS representing 2.61% of the reads code for proteins containing signatures of proteins previously associated with a protease inhibitory function, which are either ubiquitous or particular to ticks. A more detailed analysis of these transcripts follows.

#### Kunitz domain-containing proteins

Kunitz domain-containing proteins abound in tick sialotranscriptomes [2] as well as in those of the hematophagous flies of the genera *Culicoides* [54], [55] and *Simulium* [56], but not mosquitoes or sand flies. Proteins containing single or multiple Kunitz domains were described and functionally characterized in ticks, such as Ixolaris, a double-Kunitz protein, and Pentalaris, containing five domains [57], [58], [59], [60], [61], both acting as blood-clotting inhibitors of the extrinsic pathway. The Kunitz fold can also perform functions beyond protease inhibition, such as ion channel inhibition [62], [63], [64], [65]; in *R. appendiculatus*, a modified Kunitz domain peptide [66] was shown to activate maxiK channels in an *in vitro* system, suggesting a vasodilator function.

**Figure 1:**
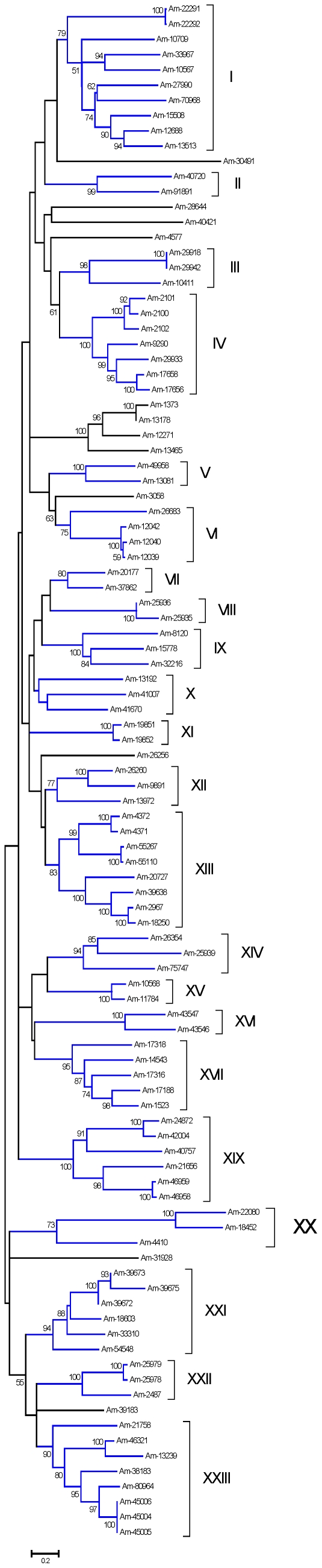
The salivary Kunitz family of proteins in *Amblyomma maculatum*. Bootstrapped phylogram (1,000 iterations) resulting from the alignment of 101 full-length protein sequences containing one or more Kunitz domains. The numbers on the nodes indicate the bootstrap support, and the bar at the bottom indicates 20% amino acid divergence. Clades with more than 70% bootstrap support are indicated by Roman numerals.

From the sialotranscriptome of *Am. maculatum*, a total of 215 CDS, extracted from 18,071 reads, were assigned to the Kunitz family of proteins based on their SMART signature. Within this group, there are proteins containing from one to six Kunitz domains. From this set, 101 are complete from starting methionine to stop codon and contain a signal peptide indicative of secretion. Several of these proteins contain one or more proP signals indicative of furin processing [45], and thus could be pro-proteins. ClustalW alignment and neighbor-joining phylogenetic analysis of this set (Figure 1) shows the diversity of this class of proteins. Gene duplication has been proposed before for explaining the expansion of protein families associated with blood feeding [11], [67]; the presence of 23 clades (defined by related proteins belonging to a clade with >70% bootstrap support) containing these 101 proteins are indicative of this process. Notice that the tree includes proteins that are very similar to each other, such as the two top sequences, or bottom three sequences, in Figure 1. These sequences could represent alleles from the same gene. It is possible that members of the same clade share the same function and are expressed at different times during feeding to avoid their host immune system. This expansion of the Kunitz family of proteins has been observed before in other tick sialotranscriptomes, but never to this degree.

#### TIL domain-containing proteins

The canonical TIL (for trypsin inhibitor-like) domain contains ten cysteines forming five disulphide bonds and is found in many protease inhibitors. These polypeptides may also exert antimicrobial function [68]. Members of this family have been found ubiquitously in bloodfeeding insect and tick sialomes, but very few have been characterized. A tick hemolymph antimicrobial peptide (AMP) was previously reported to be a member of this family [69]. More recently, tick proteins containing TIL domains were characterized from *R. microplus* as subtilisin inhibitors with antimicrobial activity and expressed in various tick organs, including the SGs [70].

**Figure 2:**
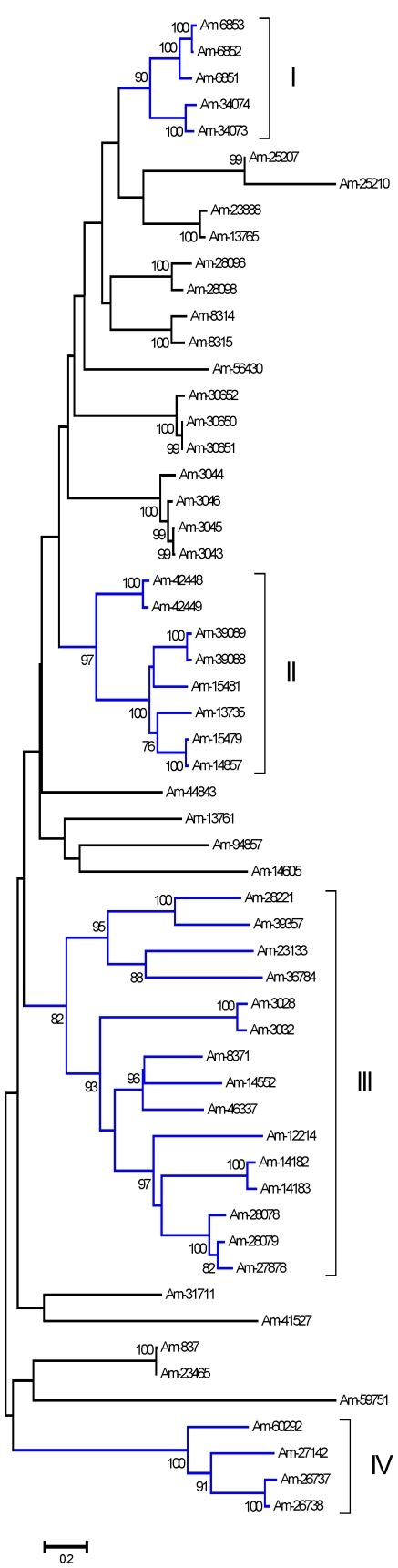
The salivary TIL domain family of proteins in *Amblyomma maculatum*. Bootstrapped phylogram (1,000 iterations) resulting from the alignment of 57 full-length protein sequences containing the Kunitz domain. The numbers on the nodes indicate the bootstrap support, and the bar at the bottom indicates 20% amino acid divergence. Clades with more than 80% bootstrap support are indicated by Roman numerals.

Eighty-five CDS were extracted from 7988 reads originating from the sialotranscriptome of Am. maculatum, 57 of which are complete from Met to stop codon and have a predicted signal peptide indicative of secretion. These proteins can contain from one to four TIL domains. Some have no TIL domain but exhibit similarities to proteins containing the domain and for this reason are included in this group. Phylogenetic analysis of the 57 related sequences (Figure 2) shows four relatively large clades with strong bootstrap support. As in the case of the Kunitz domain-containing proteins, these different TIL domain-containing proteins could have appeared as products of gene duplication, and clade members may have the same function but different antigenicities.

#### Thyropins

Thyropins are motifs found in thyroglobulins and in cysteine protease inhibitors of the actiniam-derived equistatin protein [71], [72], [73]. Equistatin has three thyropin domains, two of which were shown to be involved in protease inhibition.[73]. They are recognizable by the SMART TY domain match. A protein deducted from the sialotranscriptome of *Amblyomma variegatum* was found to have two thyropin domains [15], and one-domain proteins were found in the sialome of *R. sanguineus* [20]. Six coding sequences containing TY domains were assembled from 1,917 reads, all of which have two TY domains. Am-4121 was assembled with 1,329 reads, being the most abundantly expressed of this group, more so considering the library was normalized. All these proteins provide best matches to a *R. sanguineus* salivary thyropin deposited in the NR database, producing 54–66% identity at the amino acid sequence level. Interestingly, Am-4121 has a serine-rich carboxyterminus with 26 potential galactosylation sites, indicating it has thyropins and mucin domains. Proteins deducted from ESTs of *R. microplus* and *Am. variegatum* also have these mucin domains, and these can be inspected in the BLAST matches to the tick-tb2 database on Spreadsheet S2.

#### Cystatins

Cystatins are cysteine protease inhibitors of nearly 100 amino acids (aa) in length. Cystatins have been previously found in both hard and soft tick sialotranscriptomes. Two salivary cystatins from *I. scapularis* have been functionally characterized as inhibitors of cathepsins L and S, to inhibit inflammation, suppress dendritic cell maturation, and serve as vaccine targets [74], [75]. Twenty-five CDS were assembled from 1,100 reads originating from the *Am. maculatum* sialotranscriptome; 15 of these 25 appear full length and have a signal peptide indicative of secretion.

#### Serpins

Serpins are a ubiquitous protein family associated with the function of serine protease inhibition, from which the family name derives. A single tick salivary serpin from *I.* ricinus has been shown to inhibit vertebrate elastase and to have immunosuppressive activity. [76], [77] Another salivary serpin from the same tick inhibits cathepsin G and chymase [78]. Tick serpins have been proposed as anti-tick salivary vaccines, including non-salivary expressed serpins. [79], [80] The sialotranscriptome of *Am. maculatum* reveals 32 CDS for members of the serpin family, assembled from 1,100 reads. Eight of these CDS appear as full length and with a signal peptide indicative of secretion.

#### Kazal domain

The Kazal domain is also associated with serine protease inhibitors and antimicrobial activity [81], [82]. Members of this family have been found in anopheline and culicine mosquito sialotranscriptomes and also occasionally in tick sialotranscriptomes. In the mosquito *Aedes aegypti*, a salivary-expressed Kazal-containing peptide was shown to inhibit thrombin and plasmin with high affinity [83]. The Kazal domain can also be found in multidomain proteins, as was the case of a protein found in the sialotranscriptome of *Am. variegatum* containing an insulin growth factor binding domain in tandem with a Kazal domain, suggesting this protein could function in the modulation of signal transduction cascades.

The CDS Am-25261 is such a complex protein, containing a Kazal domain in its amino terminal region (aa 57–101), an EF-hand domain (aa 150–222), and a von Willebrand factor type C domain in the carboxyterminus (aa 232–277). This protein is 82% identical in amino acid sequence to the follistatin-related protein (FRP) of the tick *Haemaphysalis longicornis* [84]. Transcripts for this protein in *Haemaphysalis* were expressed in several organs and may indicate it has a housekeeping function; however, vertebrate FRP—which are 40% similar to the tick protein—can modulate ion transport in neurons in a way that makes them hyperpolarized and with higher excitation thresholds [85].

#### Carboxypeptidase inhibitors (CI)

**Figure 3:**
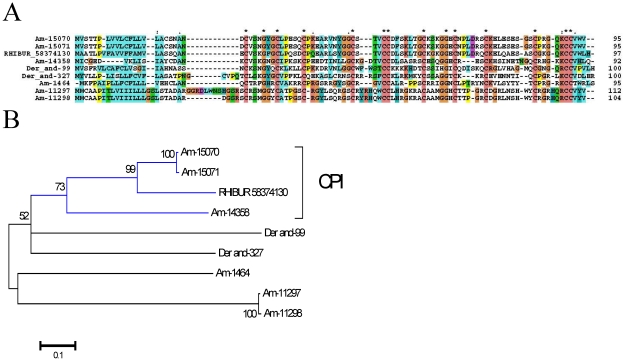
The tick salivary carboxypeptidase inhibitor family of proteins. (**A**) Alignment of *Amblyomma maculatum*, *Dermacentor andersoni*, and *Rhipicephalus bursa* proteins. (**B**) Bootstrapped phylogram (1,000 iterations) resulting from the alignment in (**A**). The numbers on the nodes indicate the bootstrap support above 50%. The clade indicated by CPI contains the functionally characterized *R. bursa* protein. The bar at the bottom indicates 10% amino acid divergence. The *Dermacentor* proteins were deducted from ESTs deposited in DBEST.

This is a protein family found to date only in metastriate ticks first identified in *R. bursa*, from where the proteins functionally characterized and crystallized [86], [87]. CI homologues from *Ha. longicornis* were also characterized [88]. These inhibitors may affect fibrinolysis by inhibiting plasma carboxypeptidase B, also known as thrombin-activatable fibrinolysis inhibitor [86]. Six CDS coding for members of this family were deducted from 425 reads derived from the sialotranscriptome of *Am. maculatum*. Alignment of these sequences with the canonical *R. bursa* protein plus *Dermacentor andersoni* sequences obtained from a previous review [2] shows 12 conserved cysteines, 4 of which are in 4 cysteine knot (CC) configuration (Figure 3A). Only three of the *Am. maculatum* proteins are within the same clade as the *R. bursa* protein (Figure 3B), indicating the diversity of this family.

#### Phosphatidylethanolamine-binding protein family

**Figure 4:**
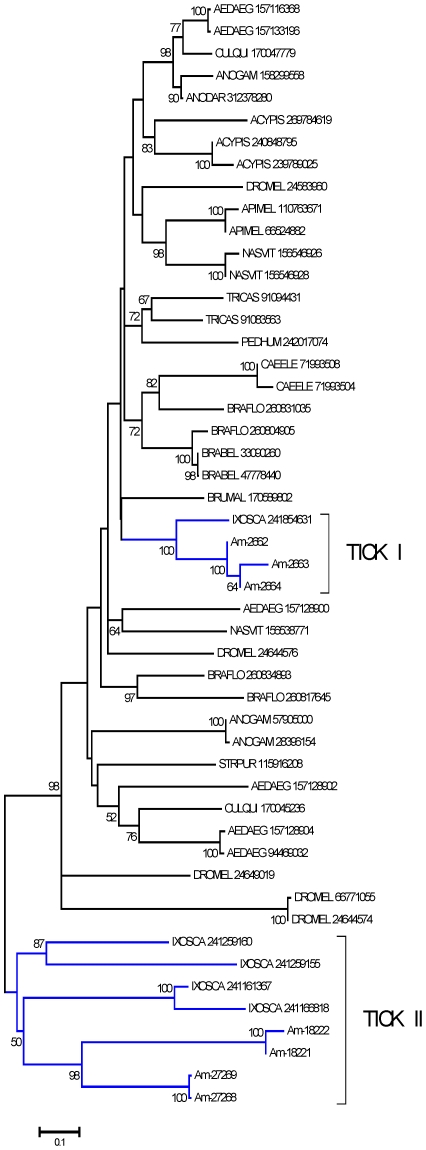
Phylogram of the phosphatidylethanolamine binding protein (PEBP) family of selected arthropods. The bootstrapped phylogram (1,000 iterations) was obtained from the alignment of deducted *Amblyomma maculatum* proteins with homologs found in the non-redundant protein database of the NCBI. The *Am. maculatum* protein names start with Am- and the remaining proteins are named by the first three letters of the genus name followed by the first three letters of the species name followed by their NCBI gi| accession number. The number at the nodes indicates the bootstrap support, and the bar at the bottom indicates 10% amino acid divergence. Two tick clades are indicated by Roman numerals.

This is a ubiquitous protein family that has been associated with serine protease inhibition [89], [90], although such activity has never been functionally characterized from any bloodsucking arthropod to date. Seven CDS were extracted from 815 reads deriving from the *Am. maculatum* sialotranscriptome. Overall, these proteins share less than 40% similarity and represent at least three expressed genes with possible alleles, as indicated by phylogenetic analysis of their closest invertebrate proteins (Figure 4), where the robust clade named TICK I has at least one gene, and the clade marked TICK II has at least 2 genes.

### Enzymes

A total of 415 CDS, representing 4% of the reads, code for proteins containing enzyme signatures and a secretion signal, or are related to secreted enzymes. Some of these enzymes could actually be destined to the ER, the Golgi apparatus, or to lysosomes and not actually secreted.

#### Proteases

**Figure 5:**
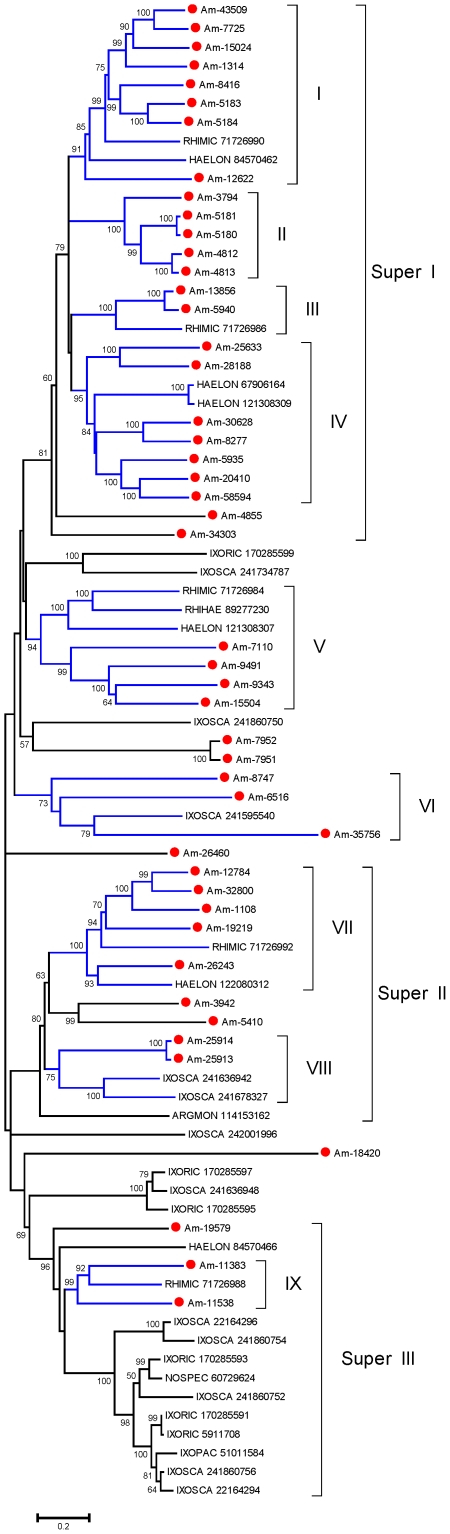
Phylogram of the tick salivary metalloproteases of the reprolysin family. The bootstrapped phylogram (1,000 iterations) was obtained from the alignment of deducted *Amblyomma maculatum* proteins with homologs found in the non-redundant protein database of the NCBI. The *Am. maculatum* protein names start with Am- and are recognized by a red circle marker; the remaining proteins are named by the first three letters of the genus name followed by the first three letters of the species name followed by their NCBI gi| accession number. The number at the nodes indicates the bootstrap support, and the bar at the bottom indicates 20% amino acid divergence. Clades and superclades with strong bootstrap support are indicated with Roman numerals.

Proteases represent the bulk of the extracted sequences coding for enzymes, represented by 311 CDS deriving from 3.2% of the reads. Among this class of enzymes, metalloproteases predominate, with 268 extracted CDS carrying 2.6% of the reads. Metalloproteases of the reprolysin family have been commonly found in tick sialotranscriptomes [2], [91], [92]. These enzymes can be identified by the CDD domain CDD|58573 cd04272, ZnMc_salivary_gland_MPs, which is quite specific for tick salivary reprolysin metalloproteases. In *I. scapularis*, metalloproteases of this type were associated with fibrinogenolytic and anti-angiogenic activities found in this tick saliva [93], [94]. *Am. maculatum* metalloproteases appear to be a highly expanded gene family, as deducted from the phylogenetic analysis of the proteins recovered with >500 aa aligned with their best matches from the NR database (Figure 5), these matches being all from ticks. Notice that there are many polyspecific clades containing *Am. maculatum* proteins in at least three super clades. This suggests the ancestral tick to prostriates and metastriates already contained at least three metalloprotease-coding genes before evolution of the ticks, with posterior gene duplication events to create the internal clades.

Metalloproteases containing the PFAM peptidase_M13_N motif include ubiquitous metalloproteases involved in peptide processing, such as the neprilysins. Neprilysins, however, are typically extracellular membrane-bound proteins of type II characterized by an uncleaved hydrophobic segment near the NH2 terminus, creating a membrane anchor [95], [96]. To the extent that tick proteins have this domain, they should not appear in tick saliva unless the tick enzymes lost their membrane-anchoring domains. The sialotranscriptome of *Am. maculatum* reveals 41 CDS producing matches to this class of enzymes, 3 of which appear to be full length (with more than 700 aa residues) and to contain a signal peptide indicative of secretion and no membrane anchor as indicated by the SignalP server [37]. For example, the tick protein coded by Am-19220 has better identity to its chicken homolog than to its *I. scapularis* homolog. While Am-19220 has a clear signal peptide indicative of secretion, the bird enzyme has a typical membrane anchor as predicted by the SignalP-HMM server [97]. Am-19220 also has, uniquely, a Kunitz domain in its amino-terminus. Am-26776 and Am-23443 are two other *Am. maculatum* neprilysins-type metalloproteases with clear signal peptide indicative of secretion. These enzymes could play a role in degrading host inflammatory peptides.

The PFAM peptidase_M2 domain identifies dipeptidyl carboxypeptidases, including the family prototype, the vertebrate angiotensin-converting enzyme. Enzymes of this family have been implicated in bradykinin degradation by the saliva of *I. scapularis* [98]. Three CDS coding for members of this family are identified in the sialotranscriptome of *Am. maculatum*, two of which appear to be full length and contain signal peptides indicative of secretion. Am-930 appears to be the most expressed, the CDS of which was assembled with 3,724 reads, more than double the reads of all neprilysins-coding CDS.

Serine proteases are commonly found expressed in arthropod sialotranscriptomes but rarely characterized for their substrate specificity. In tabanids, one such salivary enzyme was shown to have fibrinolytic activity [99]. Several serine proteases were recognized in the sialotranscriptome of *Am. maculatum*, from where 18 CDS were assembled from 2,657 reads. Six of these enzymes appear full length and with signal peptide indicative of secretion.

Proteases of the legumain family (asparaginyl endopeptidases) belong to the CD clan, family C13 of cysteine proteases, and are commonly found in tick midgut transcriptomes [100], [101], [102], [103], [104], [105], [106]. Five CDS coding for legumains were identified in the present work, three of which appear to be full length. Despite having a signal peptide indicative of secretion, these proteins could be membrane bound or directed to the lysosome. Similarly, two CDS coding for cathepsin-L type of peptidases and containing a signal peptide indicative of secretion could be destined to the lysosome.

#### Endonucleases

These enzymes cleave RNA or DNA and have been found previously in transcriptomes of mosquitoes, sand flies [107], and ticks [2], [15], but only in the mosquito *Culex quinquefasciatus* was an endonuclease shown to be secreted in saliva, and the recombinant enzyme revealed its specificity for double-stranded DNA [108]. This enzyme may help reduce the viscosity of the lacerated skin matrix but may also affect neutrophil extracellular trap formation, which is DNA rich [109]. The sialotranscriptome of *Am. maculatum* reveals several CDS coding for endonucleases, including four that appear to be full length and contain a signal peptide indicative of secretion (Am-4803, Am-12130, and Am-1109).

#### 5′-nucleotidase/apyrases

Apyrases are commonly found in saliva of bloodsucking arthropods, where they hydrolyze ATP and ADP to AMP, thus serving an antihemostatic and antiinflammatory function, because ATP and ADP—released by injuried cells, activated platelets, and neutrophils—are agonists of inflammation and platelet aggregation [2]. Mosquitoes and triatomines of the Triatoma genus have apyrases belonging to the 5′ nucleotidase family [110], [111], [112], as have soft ticks [113]. While canonical 5′ nucleotidases are extracellular enzymes bound to the cell via a glycosylphosphatidylinositol anchor, salivary apyrases lack the anchor and thus appear free in saliva [112], [114], [115].The sialotranscriptome of *Am. maculatum* reveals several members of the 5′ nucleotidase family including two relatively that are highly expressed (Am-4536 with 495 reads, and Am-9644 with 208 reads), both full length and not containing a glycosylphosphatidylinositol anchor as indicated by the Frag-anchor site (http://navet.ics.hawaii.edu/~fraganchor/NNHMM/NNHMM.html) [116]. Interestingly, these abundantly expressed apyrases share only 46% identities at the amino acid level and possibly represent a mechanism of antigenic variation.

#### Lipases and esterases

Abundant expression of mRNA coding for members of these families are found in the sialome of *Am. maculatum*, including secreted phospholipase A_2_ (Am-9375 with 636 reads), a glycosylphosphatidylinositol-specific phospholipase (Am-14132 with 592 reads), and a sphyngomyelinase (Am-21256 with 527 reads). These enzymes may affect host signaling pathways in inflammation and immunity.

#### Glycosidases

Several contigs coding for glycosidases with signal peptide indicative of secretion were found in the sialotranscriptome, but they are most probably lysosomal enzymes and are not particularly highly expressed, with less than 40 reads on the most abundant contigs. We call particular attention to Am-23781, which is a truncated contig having the hyaluronoglucosaminidase KOG domain. Hyaluronidases have been described in hematophagous flies [99], [117], [118], [119] and in the saliva of *Amblyomma hebraeum* [120], where they may serve to break down the host skin matrix and also affect chemokine signaling, which needs the negatively charged matrix of sulfated glycan acid for its function [121], [122].

#### Sulfatases

Two sulfatases with signal peptide indicative of secretion were found in the sialotranscriptome. These enzymes are normally lysosomal but, if secreted, they could have a role in degrading skin mucopolysacharides, as indicated in the previous section.

### Lipocalins

The lipocalin family is widespread in nature and is abundantly expressed in ticks and triatomine sialotranscriptomes as members of a large gene family. In *Am. maculatum*, we were able to extract a total of 584 coding sequences from this family with 5 or more reads, 55 of which have more than 300 reads, indicating their relatively abundant expression. Lipocalins may function as anticomplement [123], but more widely as scavengers or kratagonists [124] of biogenic amines and arachiconid acid-derived agonists of hemostasis and inflammation such as thromboxane A_2_ and leukotrienes [125], [126], [127], [128], [129], [130]. Seven of the *Am. maculatum* CDS coding for lipocalins were assembled from more than 1000 reads. These abundant lipocalins may be kratagonists of serotonin or histamine, because these agonists accumulate to near micromolar amounts during inflammation and hemostasis in contrast with nanomolar ranges of concentration for leukotrienes. It is quite remarkable that these lipocalins from *Am. maculatum* are at best only 40% identical to other tick lipocalins, suggesting a fast evolution of this protein family.

### Antigen 5/CRISP/CAP

This protein family is ubiquitously found in plants and animals, having being found in the venom of vespids (whence the name antigen 5 comes [131]); in snake venoms, where they are known as cysteine rich secretory protein family (thus the name CRISP) and have toxic properties [132], [133], [134]; and in plants, where they are associated with pathogen responses. The superfamily CAP recovers the families known as CRISP, antigen 5, and pathogen-associated proteins from plants [135]. Virtually all sialotranscriptomes of bloodsucking arthropods have members of this family [2], [124]. In stable flies, a member of the family binds immunoglobulins and may inhibit the classical pathway of complement [136], [137]; an antigen 5 protein from tabanids uniquely inhibits platelet aggregation and angiogenesis [99], [138], [139] through acquisition of an RGD (disintegrin) domain [140]. The sialotranscriptome of *Am. maculatum* revealed several members of this family. Spreadsheet S2 presents seven coding sequences, mostly truncated.

### Prokineticin domain-containing peptides

The sialotranscriptome of ticks has revealed proteins with the PFAM prokineticin domain. Prokineticins are peptides secreted by the suprachiasmatic nucleus of mammals and associated with circadian rhythm (http://pfam.sanger.ac.uk/family?acc=PF06607). Interestingly, a protein inducing smooth muscle contraction from the venom of the black mamba snake is a member of this family [141]. Four full-length CDS from this family were extracted from the sialotranscriptome of *Am. maculatum*, three of which are more than 20% divergent from each other and may derive from different genes. No function is known for any of the tick prokineticin domain salivary proteins.

### Serum amyloid domain-containing proteins

In mammals, this protein family is a marker of acute-phase response and has been associated with endothelial function and tumor growth [142], [143]. Members of this family have been found in previous sialotranscriptome of ticks, but none of their functions is known. Four full-length CDS have been retrieved from the sialotranscriptome of *Am. maculatum*, three of which appear to be alleles of the same gene.

### Mucins

These are serine/threonine-rich proteins that can be heavily glycosylated with N-acetyl galactosamine residues [144], [145]. They are commonly found in mucosal tissues, where they serve a role in mechanical protection of the cells and in preventing pathogen invasion. Their primary structure can be diverse; tick proteins of this family do not share significant sequence similarities to non-tick proteins except for the pattern of some repeated amino acids. Their galactosylation sites can be recognized by the site NetOGlyc [146]. Am-17147, constructed from 489 reads, has a signal peptide indicative of secretion and 58 glycosylation sites predicted by the NetOGlyc server. It shares only 29% identity to its *I. scapularis* homolog. Fifteen other putative mucins are indicated in Spreadsheet S2.

### Secreted immunity-related products

Antimicrobial peptides of the defensin, hebreain/microplusin/Ricinusin, and lysozyme families [147] are represented in Spreadsheet S2, as well as peptides weakly similar to plant antimicrobials. Pathogen-recognition proteins of the peptidoglycan-recognition family, of the ixoderin/ficolin family of fibrinogen-domain containing proteins [148], [149], [150], and of the ML domain family [2], [151] are also represented.

### Tick-specific protein families

The sialotranscriptome of ticks abound with glycine-rich proteins, many of which were identified as members of the salivary cement used to attach the tick to its host's skin [152], [153], [154], [155]. Members of the Ixodes genus also contain collagen-like glycine rich proteins. Additionally, cuticle and perithrophin proteins as well as some classes of antimicrobial peptides are rich in glycine and are included in this broad class [2]. We call attention to a large gene expansion indicated by the family GRP 40-21 (Spreadsheet S2), where 23 CDS were extracted, including Am-2829 obtained from 1184 reads, which may represent the most abundant cement protein of *Am. maculatum*. This protein is only 38% identical to an *Am. variegatum* homolog, indicating a fast evolution of this multigene family. Another group of proteins, both glycine and tyrosine rich, belongs to the previously named large GGY family [2]. Am-1905 is a highly expressed member of this family, being assembled from 1,932 reads.

#### Ixodegrins

This tick family, members of which range from 100–200 aa, was initially identified in *I. pacificus* [156] and found to be abundantly expressed in tick sialotranscriptomes [2]. A reverse position matrix made from the alignment of homologous proteins obtained from previous sialotranscriptomes was used by the BLAST package tool rpsblast to identify *Am. maculatum* members of this family, 20 of which are shown in Spreadsheet S2. Some of these proteins have weak matches to the PFAM prokineticin domain, as well as to the colipase domain, indicating Ixodegrins may be related to the prokineticin-domain proteins identified above. No member of this family has been functionally characterized thus far.

#### Disintegrins

**Figure 6:**
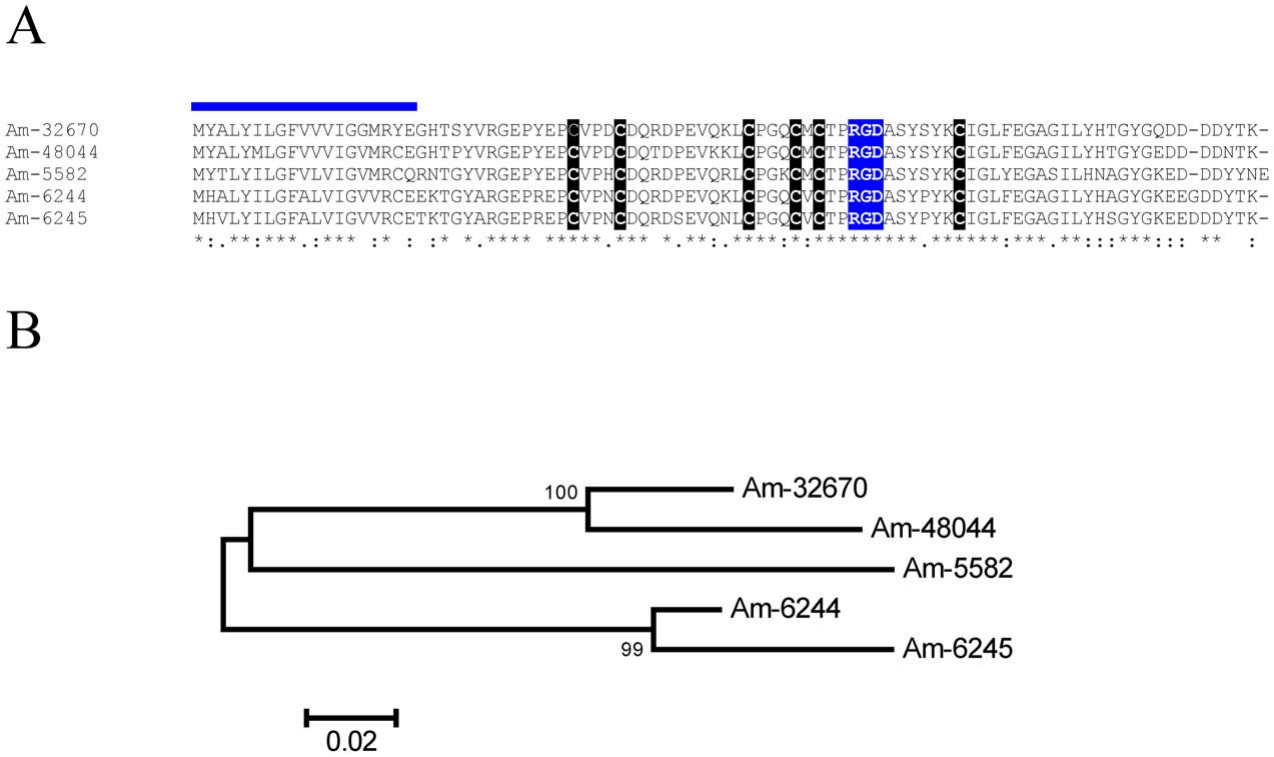
The salivary disintegrins 40–270 family of *Amblyomma maculatum*. (**A**) Alignment indicating conserved cysteines in black background, RGD motif in blue background, and blue bar above the sequences indicating the signal peptide indicative of secretion. The symbols at the bottom indicate (*) identity of residues, (:) conserved, and (.) less conserved residues. (**B**) Bootstrapped phylogram of the alignment in (**A**). The numbers at the nodes indicate the bootstrap support, and the bar at the bottom indicates 2% amino acid divergence.

This name derives from snake venom toxins having the RGD domain flanked by cysteines, which mimics the fibrinogen motif that binds to their platelet integrin receptor. Disintegrins thus occupy the integrin site destined for fibrinogen in the platelet surface, preventing platelet aggregation, because aggregation of platelets occurs by crosslinking of platelets by fibrinogen, which has multiple RGD sites [1], [140], [157]. Variations of the RGD domain exist, including KGD, KTS, and RTS domains [158]. The sialotranscriptome of *Am. maculatum* identified several members of this family, including five members of the related but divergent disintegrin 40–270 family (Figure 6A). Phylogenetic analysis is indicative of at least three genes coding for this family (Figure 6). Spreadsheet S2 displays additional polypeptides containing the RGD motif as well as the RTS motif.

#### Salp15

A salivary protein from *I. scapularis* was shown to inhibit CD4^+^ T cell activation and to be important for Lyme's disease transmission [5], [159], [160], [161], [162]. A PFAM domain from this family (PF12115), constructed from *Ixodes* and *Argas* proteins, identified ten very divergent members of this family in *Am. maculatum* with relatively high E values. Members of this family have not been found in metastriate ticks previously. These ten *Am. maculatum* members can be divided into four families, containing four, two, two, and two proteins each, coding for proteins of mature size ranging from 10 to 26 kDa (Spreadsheet S2). The family with larger molecular weight (MW) has many galactosylation sites indicative of mucin.

#### Basic tail/18.3-kDa superfamily

This superfamily was identified in *I. scapularis* and then found to abound in other tick sialotranscriptomes. The “basic tail” name derives from a cluster of basic amino acids on the carboxyterminus of the protein. This basic tail may direct the protein to negatively charged phospholipids [163], [164] that serve as scaffolding for the proteinase complexes that activate clotting [1]. The 18.3-kDa family does not have this basic tail, but was identified by using the tool PSI-BLAST searching the NR database starting with a basic tail protein. A PFAM domain named TSGP1 helps to identify both members of the family. Spreadsheet S2 identifies 40 members of this superfamily, either having the PFAM TSGP1 domain, or having similarities to members of the superfamily. Most CDS are full length and with a signal peptide indicative of secretion. Several of these sequences have indication of abundant galactosylation, indicating that they might serve as mucins. Only one member of the family, named salp9, has been functionally characterized as an anticlotting protein [165].

#### 23-kDa family

**Figure 7:**
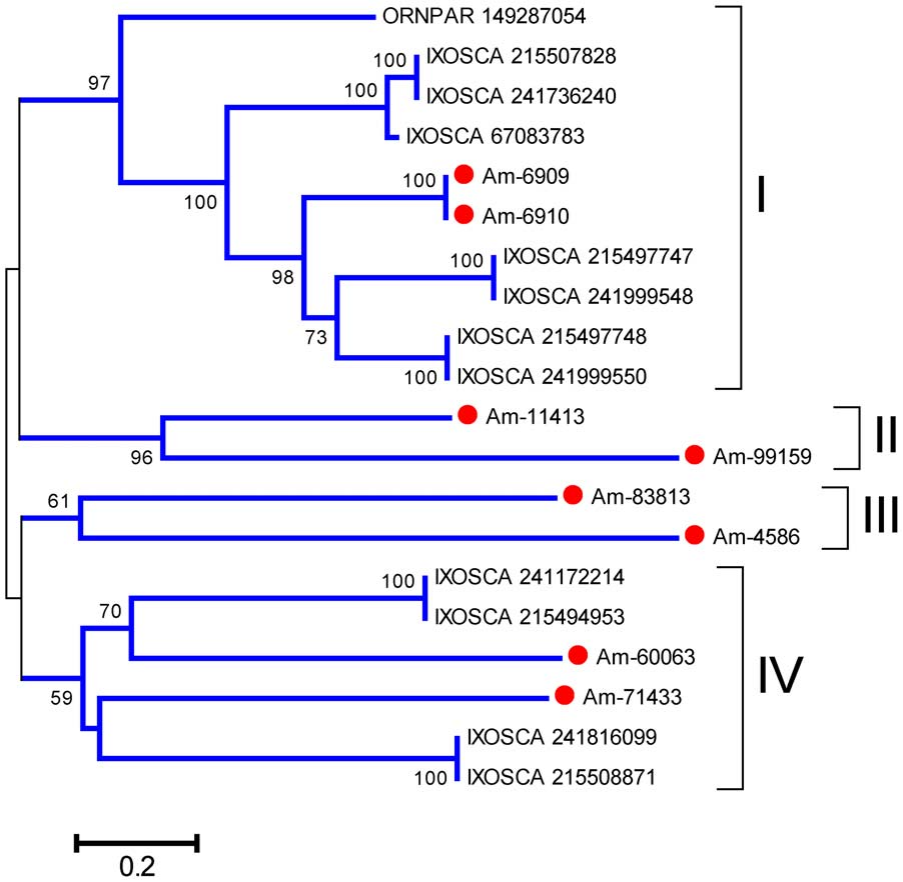
Phylogram of tick proteins from the 23-kDa family. The bootstrapped phylogram (1,000 iterations) was obtained from the alignment of deducted *Amblyomma maculatum* proteins with homologs found in the non-redundant protein database of the NCBI. The *Am. maculatum* protein names start with Am- and are recognized by a red circle marker; the remaining proteins are named by the first three letters of the genus name followed by the first three letters of the species name followed by their NCBI gi| accession number. The number at the nodes indicates the bootstrap support above 50%, and the bar at the bottom indicates 20% amino acid divergence. Clades and superclades with strong bootstrap support are indicated with Roman numerals.

This protein family has, exclusive of soft and hard ticks [2], no known function. The sialotranscriptome of *Am. maculatum* identifies eight members of the family, as revealed by rpsblast using a model based on the previously known sequences (Additional Spreadsheet S2). Phylogenetic analysis of these proteins resulting from their alignment with tick proteins found in the NR database by BLAST search with an E value smaller than 1e-20 (Figure 7) shows strong support for a clade containing several *I. scapularis* proteins, one *Ornithodoros* protein, and two very similar *Am. maculatum* proteins, possible alleles (Figure 7, Clade I). Additional clades indicate another six divergent genes coding for this protein family in *Am. maculatum*. Messages for Am-6910 and Am-4586 are abundant, the contigs having been assembled with over 400 reads.

#### 8.9 kDa family

This is a protein family, exclusive of hard ticks, coding for secreted peptides of mature MW near 10 kDa, but also having proteins of double the size containing two domains. Their function is unknown. Coding sequences for 83 members of this family are reported for *Am. maculatum* in Spreadsheet S2. Some members of this family in *Am. maculatum* are abundantly expressed, their CDS being assembled with over 1,000 reads (Am-3961 and Am-733). Phylogenetic analysis of these proteins with their best matches from the NR database is complex, showing robust clades with both prostriate and metastriate sequences as well as clades showing expansion of *Am. maculatum* genes (not shown), suggestive of multiple rounds of gene duplication and fast evolution of this family.

#### Ixostatin

Ixostatins are an expanded protein family found in *I.s scapularis* and *I. pacificus* [12], [156], but a few members were found in *Dermacentor* and *Amblyomma* [2]. Three members of this family are found in *Am. maculatum*, all poorly expressed, their CDS being assembled from 11–40 reads.

#### One-of-each family

This family was so named because only one protein per tick species has been found in sialotranscriptomes [2]; however, 24 CDS coding for members of this family were found in the sialotranscriptome of *Am. maculatum*.

**Figure 8:**
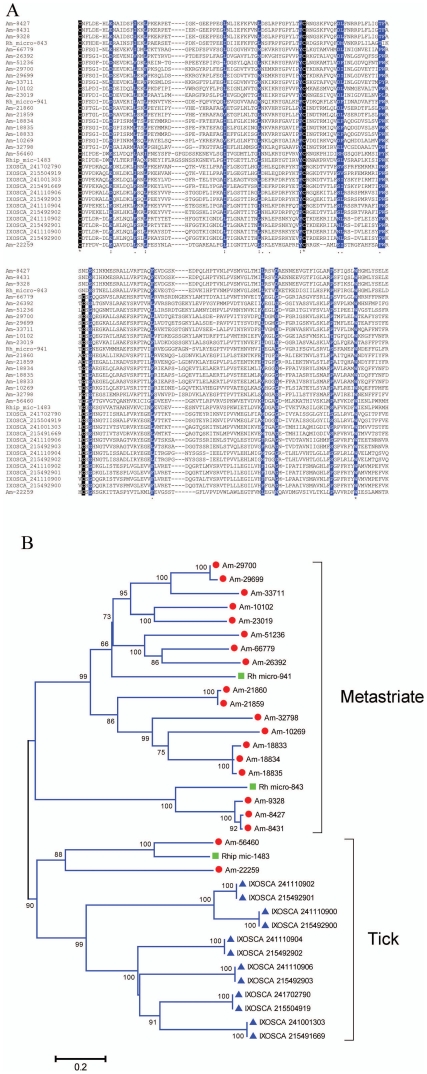
The one-of-each family of ticks. (**A**) Alignment indicating conserved cysteines in black background, conserved residues in blue background. Sequences starting with Am are from *A. maculatum* and are recognized by a red circle, those starting with IXOSCA are from *Ixodes scapularis* retrieved from GenBank and are recognized by a blue triangle, those from *Rhipicephalus* were assembled from DBEST ESTs as described before and are recognized by a green square [1]. The symbols at the bottom indicate (*) identity of residues, (:) conserved and (.) less conserved residues. (**B**) Bootstrapped phylogram of the alignment in (**A**). The numbers at the nodes indicate the bootstrap support and the bar at the bottom indicates 20% amino acid divergence.

PSI-BLAST search of the NR database using Am-18834 as query retrieves only tick proteins, indicating the specificity of the family (Additionalal file S3); however, several *I. scapularis* additional members of this family were found, indicating it to be a multigene family, as it appears to be in *Am. maculatum*. Alignment of members of the family having more than 200 aa indicates a very diverse family with few conserved amino acids (Figure 8A). The phylogram shows two robust superclades—one with *Amblyoma* and *Rhipicephalus* sequences and another with metastriate and prostriate proteins (Figure 8B)—and indicate at least 14 genes coding for this family in *Am. maculatum* (indicated by sequences >20% distant from each other) and 5 for *I. scapularis*.

#### Novel family 40-33

**Figure 9:**
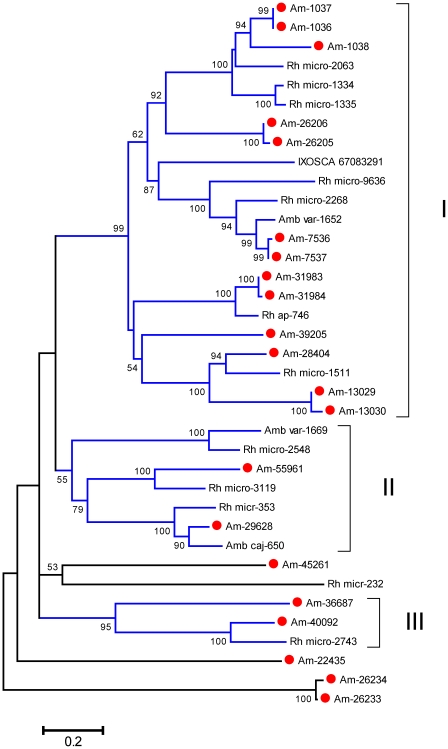
Phylogram of tick proteins from the novel 40-33 family. The bootstrapped phylogram (1,000 iterations) was obtained from the alignment of deducted *Amblyomma maculatum* proteins with homologs deducted from DBEST sequences described in a previous publication [2]. The *Am. maculatum* protein names start with Am- and are recognized by a red circle marker; the *Ixodes scapularis* protein is named IXOSCA followed by the first three letters of the species name followed by their NCBI gi| accession number. The number at the nodes indicates the bootstrap support above 50%, and the bar at the bottom indicates 20% amino acid divergence. Clades are indicated with Roman numerals.

The sialotranscriptome of *Am. maculatum* reveals 21 CDS that are related at the 40% identity level, constituting the 33rd most abundant cluster at this level of protein clusterization. The predicted peptides have mature MW near 22 kDa. PSI-BLAST of Am-1037 against the NR database retrieves, after five iterations, spider and acari proteins, and at later iterations retrieves also *Daphnia* and insect proteins, indicating this to be an unknown arthropod family (Additional file S4). Phylogenetic analysis of the alignment of the *Am. maculatum* members of this family with previously described tick proteins obtained from assembly of DBEST ESTs from ticks [2], as well as *I. scapularis* sequences deposited in GenBank, reveals several clades containing sequences from multiple tick species (indicated by Roman numerals in Figure 9), including clade I with strong bootstrap support and containing both metastriate and prostriate proteins. Clades II and III have metastriate proteins only. There are possibly 14 genes coding for this family in *Am. maculatum*, based on divergences larger than 20% amino acid sequence. Four members of this family in *Am. maculatum* were assembled with more than 300 reads, indicating relatively high expression levels.

#### Similar to Rhipicephalus and Ixodes proteins

Eight coding sequences from *Am. maculatum* sialotranscriptome present similarities with deducted protein sequences from *Rhipicephalus appendiculatus* deducted from ESTs deposited in DBEST, and a weaker match to an *I. scapularis* sequence deposited in GenBank. No similarities are found for other proteins in the NR database, thus characterizing a novel tick protein family.

### Metastriate-specific protein families

#### Evasins

**Figure 10:**
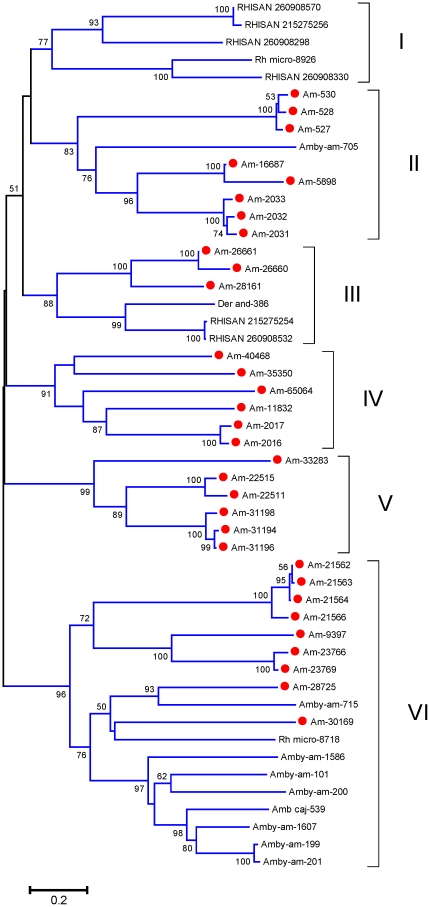
The evasin family of metastriate ticks. The bootstrapped phylogram (1,000 iterations) was obtained from the alignment of deducted *Amblyomma maculatum* proteins with homologs deducted from DBEST sequences described in a previous publication [2], and the *R. sanguineus* evasins from GenBank. The *Am. maculatum* protein names start with Am- and are recognized by a red circle marker. Other name conventions are as described in the previous figures. The number at the nodes indicates the bootstrap support above 50%, and the bar at the bottom indicates 20% amino acid divergence. Clades and superclades with strong bootstrap support are indicated with Roman numerals.

These are chemokine-binding proteins previously identified from the tick *R. sanguineus* [166], [167]. Thirty-eight CDS from *Am. maculatum* were identified as similar to evasins based on similarity matches by BLAST or by rpsblast using evasin reverse-position matrices. Phylogenetic analysis (Figure 10) indicates the diversity of the family, even within the canonical *R. sanguineus* sequences, which are grouped in clades I and III. The *Am. maculatum* sequences are found in several clades, including in clade III. Analysis indicates at least 18 different genes coding for members of this family within *Am. maculatum*, based on sequences that are >20% divergent at the amino acid level. Relatively to other CDS from *Am. maculatum*, this family is not particularly highly expressed, the largest number of reads being 247 for Am-9397. If these proteins in *Am. maculatum* function as chemokine binders, they need not be at high concentrations, as these host proteins occur at nanomolar concentrations [121].

#### DAP-36 immunosuppressant

A salivary protein of 36 kDA was previously identified as an immunosuppressant from the tick *Dermacentor andersoni* [168], [169] and found to be divergent and widespread in metastriate ticks [2]. Several members of this family are found in *Am. maculatum*, including two CDS that were assembled with more than 500 reads (Am-7601 and Am-5452). Sequence comparisons indicate at least eight genes coding for members of this family in *Am. maculatum* (data not shown).

#### Metastriate 13-kDa family

Fifteen related CDS coding for proteins with mature MW, mostly from 10–11 kDa, were found in the sialotranscriptome of *Am. maculatum* that match proteins previously classified under this name [2]. The function of these proteins is unknown.

### Deorphanized metastriate-specific protein families

**Figure 11:**
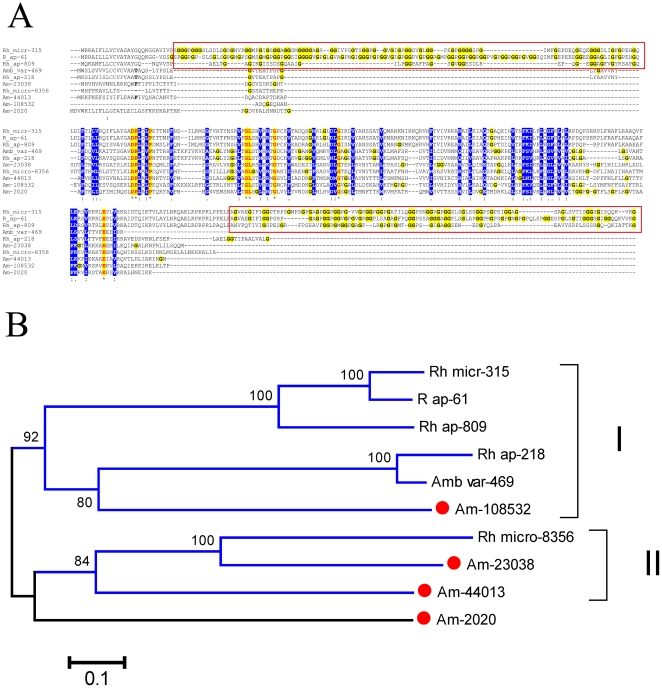
The salivary 40–279 novel family of metastriate ticks. (**A**) Clustal alignment. The symbols at the bottom indicate (*) identity of residues, (:) conserved and (.) less conserved residues. Identical residues are represented with red letters and yellow background. Conserved residues are shown in blue background. Glycines are shown in yellow background. The red boxes mark the glycine rich regions of 3 *Rhipicephalus* sequences. (**B**) Bootstraped phylogram of the alignment in A. The numbers at the nodes indicate the bootstrap support and the bar at the bottom indicates 10% amino acid divergence. The *Amblyomma maculatum* protein names start with Am- and are recognized by a red circle marker. Remaining sequences are from *Rhipicephalus microplus*, *R. appendiculatus* and *A. variegatum*. Clades with strong bootstrap support are indicated with Roman numerals.

The sialotranscriptome of *Am. maculatum* allowed the identification of 12 clusters of sequences that match previously metastriate orphan proteins (orphan proteins being defined as those without primary sequence similarity when compared to the NR protein database of NCBI by blastp with the complexity filter turned off), thus characterizing 12 novel tick protein families. We will here analyze a single family, the metastriate novel family 40–279 (Spreadsheet S2), composed of four related CDS from *A. maculatum*. Alignment of the deducted protein sequences with their matches to a tick salivary protein database [2] shows that the proteins from *Rhipicephalus* have two glycine/proline-rich regions at either end of the sequence, and a middle part that has several sites of conservation among all the sequences (Figure 11A). The phylogram (Figure 11B) shows two clades with strong bootstrap support, indicating the existence of at least four genes for *Am. maculatum*, two for *R. microplus*, and three for *R. appendiculatus*. The conserved region allows the identification of a conserved amino acid block, [FL]-x(2)-[LVMI]-[VLI]-x(9)-D-P-[LM]-x-[LVI]-P-x(18,24)-[VL]-x-G-L-x(2)-[VML]-x-[RK]-x-G-x(15)-[DN]-[LVM]-G-x(3)-[VL]-x(7)-[VLIM]-x(18,21)-[RQH]-x(2)-[LV]-x(3)-[QE]-x(6,7)-[VL]-x(2)-[FL]-x-[IVL]-x(2)-[LF]-x(4)-[LV]-x-[LVI]-x(12,21)-[LF]-x(3)-[VLI]-x(4)-E-x(2)-[VIL], that might help to identify these proteins. AM-2020 is well expressed, with 435 reads.

### Amblyomma-specific families

Three hundred seven CDS, grouped in 54 families, provide significant matches solely to previously described *Amblyomma* proteins or to other proteins deducted from the *Am. maculatum* sialotranscriptome. Spreadsheet S2 contains 1,118 CDS coding for putative secreted proteins that are not classified. With additional tick genome and transcriptome sequencing, these proteins should be deorphanized.

### Conclusions

This catalogue of salivary transcripts of *Amblyomma maculatum* herein reported represents a revolution since tick sialotranscriptomes were done 9 years ago for the first time [13], [14]. The current assembly of over 1.5 million sequences from a normalized library, instead of thousands of sequences, allowed for a depth of transcript coverage thus far unequalled in sialotranscriptomes, and the deposition of 4,850 sequences to GenBank, as compared to very few in those pioneer publications. A total of 3,475 contigs were associated with a secretory function, and these were classified into 133 families. Twelve previously orphan metastriate salivary protein families were deorphanized with the current transcriptome; 55 families were identified as *Amblyomma* specific, until other transcriptomes may deorphanize them or confirm their unique status. This data set will serve as a platform for mining new pharmacologically active proteins and for development of anti tick vaccines to deter tick feeding or the pathogens they transmit.

## Supporting Information

Table S1 (DOC)

Spreadsheet S1 (TXT)

Spreadsheet S2 (XLSX)
