# Supplementary material for: A Deep Insight into the Sialotranscriptome of the Gulf Coast Tick, Amblyomma maculatum
Source: PLoS One. 2011 Dec 21;6(12):e28525. doi: 10.1371/journal.pone.0028525 (PMC3244413; doi:10.1371/journal.pone.0028525)
Supplement: Table S1 — (DOC) [file pone.0028525.s001.doc]

**Supplemental table S1.** Functional classification of extracted coding sequences from the *Amblyomma maculatum* sialotranscriptome

| **Class** | **Number of coding sequences** | **Total reads** | **Percent of total reads** |
| --- | --- | --- | --- |
| **Putative housekeeping proteins** |  |  |  |
| Immunity related products - possibly endogenous function |  |  |  |
| Toll-like receptors | 14 | 887 | 0.07 |
| Interleukin 17-like | 3 | 55 | 0.00 |
| Similar to Interferon alpha-inducible protein | 6 | 445 | 0.04 |
| Similar to anti-viral guanylate-binding protein | 5 | 96 | 0.01 |
| Thioester complement like-protein and other complement cascade proteins | 8 | 1065 | 0.09 |
| Other proteins possibly associated with immunity | 39 | 2714 | 0.22 |
| Protein export machinery | 395 | 52361 | 4.18 |
| Cytoskeletal | 209 | 22156 | 1.77 |
| Detoxification |  |  |  |
| Sulfotransferases | 6 | 250 | 0.02 |
| Glutathione transferase | 46 | 5253 | 0.42 |
| Dehydrogenases | 29 | 1889 | 0.15 |
| O-Methyl transferases | 11 | 368 | 0.03 |
| Multidrug resistance transporters | 10 | 244 | 0.02 |
| Oxidative detoxification | 99 | 5947 | 0.48 |
| Other detoxification proteins | 29 | 2706 | 0.22 |
| Extracellular matrix and adhesion | 143 | 13198 | 1.05 |
| Amino acid metabolism | 128 | 13221 | 1.06 |
| Carbohydrate metabolism | 236 | 23323 | 1.86 |
| Energy metabolism | 251 | 39465 | 3.15 |
| Intermediate metabolism | 123 | 10879 | 0.87 |
| Lipid metabolism | 344 | 29821 | 2.38 |
| Nucleotide metabolism | 158 | 18672 | 1.49 |
| Nuclear export | 15 | 1240 | 0.10 |
| Nuclear regulation | 309 | 26889 | 2.15 |
| Protein modification machinery | 676 | 67359 | 5.38 |
| Proteasome machinery | 192 | 27612 | 2.21 |
| Protein synthesis machinery | 542 | 82422 | 6.58 |
| Signal transduction | 757 | 61883 | 4.94 |
| Transcription factors | 164 | 11217 | 0.90 |
| Transcription machinery | 686 | 69673 | 5.57 |
| Transporters and storage | 327 | 27709 | 2.21 |
| Unknown conserved proteins | 1471 | 137029 | 10.95 |
| Unknown conserved secreted proteins | 53 | 6788 | 0.54 |
| Tick-specific conserved proteins (similarity only to *Ixodes scapularis* by blastp 1e-15) | 372 | 22711 | 1.81 |
| **Putative secreted proteins** |  |  |  |
| ***Ubiquitous protein domains*** |  |  |  |
| Proteinase inhibitor domains |  |  |  |
| Kunitz domains |  |  |  |
| Hexalaris | 2 | 187 | 0.01 |
| Pentalaris | 3 | 703 | 0.06 |
| Tetralaris | 8 | 2022 | 0.16 |
| Trilaris | 8 | 1377 | 0.11 |
| Bilaris | 67 | 6089 | 0.49 |
| Monolaris | 127 | 7693 | 0.61 |
| TIL domain (may have anti-microbial activity) | 85 | 7988 | 0.64 |
| Thyropin domains | 6 | 1520 | 0.12 |
| Cystatin | 25 | 1100 | 0.09 |
| Serpin | 32 | 2124 | 0.17 |
| Kazal domain containing peptides | 2 | 272 | 0.02 |
| Carboxypeptidase inhibitor | 6 | 425 | 0.03 |
| Phosphatidylethanolamine-binding protein | 7 | 815 | 0.07 |
| ***Enzymes*** |  |  |  |
| Secreted peptidases |  |  |  |
| Metalloproteases of the reprolysin family | 210 | 26979 | 2.15 |
| Neprilysin-type metalloprotease | 42 | 3542 | 0.28 |
| Other metalloproteases | 39 | 1426 | 0.11 |
| Dipeptidyl-peptidase | 3 | 4010 | 0.32 |
| Serine proteases | 18 | 2657 | 0.21 |
| Legumain family | 5 | 284 | 0.02 |
| Papain family - possible lysosomal | 2 | 916 | 0.07 |
| Other proteases | 14 | 1418 | 0.11 |
| Secreted nucleases |  |  |  |
| Deoxyribonuclease II | 14 | 1090 | 0.09 |
| dsRNA-specific ribonuclease | 6 | 475 | 0.04 |
| Ribonuclease, T2 family | 2 | 195 | 0.02 |
| Other endonucleases | 6 | 354 | 0.03 |
| 5' nucleotidase/apyrase | 9 | 971 | 0.08 |
| Lipases and esterases | 51 | 5977 | 0.48 |
| Secreted glycosidases | 23 | 562 | 0.04 |
| Sulfatases | 2 | 43 | 0.00 |
| Lipocalins | 584 | 70938 | 5.67 |
| Antigen 5 family | 7 | 779 | 0.06 |
| Prokineticin domain peptides | 4 | 996 | 0.08 |
| Serum amyloid family | 4 | 276 | 0.02 |
| Mucins | 16 | 1672 | 0.13 |
| ***Secreted immunity related proteins*** |  |  |  |
| Antimicrobial peptides |  |  |  |
| Defensins | 13 | 243 | 0.02 |
| Hebreain/Ricinusin/Microplusin family | 20 | 949 | 0.08 |
| Lysozyme | 4 | 93 | 0.01 |
| Similar to Ipomoea amp | 3 | 84 | 0.01 |
| Vicilin-like antimicrobial peptides | 1 | 6 | 0.00 |
| Pathogen recognition proteins |  |  |  |
| Peptidoglycan recognition protein | 8 | 516 | 0.04 |
| Ixoderin | 10 | 381 | 0.03 |
| ML - Niemann-Pick family | 25 | 1990 | 0.16 |
| ***Tick specific proteins*** |  |  |  |
| Glycine rich family |  |  |  |
| Cuticle like proteins | 18 | 384 | 0.03 |
| Peritrofins | 10 | 1283 | 0.10 |
| GRP 40-21 | 23 | 3674 | 0.29 |
| GRP 40-85 | 4 | 213 | 0.02 |
| YYYGR | 7 | 171 | 0.01 |
| Large GGY protein group II | 4 | 2158 | 0.17 |
| Other Gly or proline rich proteins | 56 | 4227 | 0.34 |
| Ixodegrins | 20 | 1525 | 0.12 |
| Disintegrins |  |  |  |
| RGD-40-207 family | 5 | 403 | 0.03 |
| RTS disintegrin | 4 | 74 | 0.01 |
| Other RGD containing peptides | 6 | 199 | 0.02 |
| Salp-15 family | 10 | 432 | 0.03 |
| Basic tail 18.3 superfamily | 40 | 5109 | 0.41 |
| 23 kDa family of tick proteins | 8 | 1343 | 0.11 |
| 8.9 kDa family | 79 | 13390 | 1.07 |
| Ixostatin-like | 3 | 81 | 0.01 |
| One-of-each family | 24 | 2860 | 0.23 |
| Novel family 40-33 | 21 | 2631 | 0.21 |
| Similar to Rhipicephalus and Ixodes proteins | 8 | 311 | 0.02 |
| ***Metastriate specific proteins*** |  |  |  |
| Evasins | 38 | 2302 | 0.18 |
| Evasin group 2 | 5 | 409 | 0.03 |
| Evasin group 3 | 6 | 440 | 0.04 |
| DAP-36 immunosuppressant family | 14 | 2132 | 0.17 |
| Metastriate 13 kDa family | 15 | 653 | 0.05 |

| ***Deorphanized metastriate protein families*** |  |  |  |
| --- | --- | --- | --- |
| Metastriate novel family 40-279 | 4 | 500 | 0.04 |
| Similar to Hyaloma gi|307006465 protein | 17 | 1151 | 0.09 |
| Similar to Rhipicephalus gi|260908580 protein | 16 | 1027 | 0.08 |
| Metastriate specific protein family 40-221 | 5 | 60 | 0.00 |
| Metastriate specific protein family 40-173 | 5 | 511 | 0.04 |
| Metastriate Cys-rich specific protein family 40-162 | 8 | 324 | 0.03 |
| Metastriate specific protein family 40-93 | 10 | 818 | 0.07 |
| Metastriate specific protein family 40-49 | 16 | 2150 | 0.17 |
| Family 40-276 | 3 | 180 | 0.01 |
| Metastriate specific protein family 40-222 | 5 | 268 | 0.02 |
| Metastriate specific protein family 40-314 | 4 | 481 | 0.04 |
| Metastriate specific protein family 40-109 | 6 | 181 | 0.01 |
| ***Amblyomma-specific proteins*** |  |  |  |
| Simillar to platelet endothelial cell receptor | 3 | 56 | 0.00 |
| Amblyoma specific protein family 40-579 | 3 | 300 | 0.02 |
| Amblyoma specific protein family 40-597 | 2 | 61 | 0.00 |
| Amblyoma specific protein family 40-610 | 3 | 327 | 0.03 |
| Amblyoma specific protein family 35-28 | 21 | 7805 | 0.62 |
| Amblyoma specific protein family 35-33 | 15 | 1041 | 0.08 |
| Amblyoma specific protein family 40-123 | 5 | 154 | 0.01 |
| Amblyoma specific protein family 40-102 | 9 | 282 | 0.02 |
| Amblyoma specific protein family 35-217 | 3 | 27 | 0.00 |
| Amblyoma specific protein family 40-303 | 2 | 668 | 0.05 |
| Amblyoma specific protein family 40-484 | 3 | 88 | 0.01 |
| Ricin-like | 2 | 16 | 0.00 |
| Amblyoma specific protein family 40-271 | 3 | 314 | 0.03 |
| Amblyoma specific protein family 40-138 | 3 | 995 | 0.08 |
| Amblyoma specific protein family 35-165 | 4 | 305 | 0.02 |
| Amblyoma specific protein family 35-165 | 3 | 119 | 0.01 |
| Amblyoma specific protein family 40-321 | 4 | 162 | 0.01 |
| Amblyoma Histidine rich protein | 2 | 262 | 0.02 |
| Amblyoma cysteine rich protein family 40-288 | 5 | 118 | 0.01 |
| Amblyoma 9 kDa family | 2 | 31 | 0.00 |
| Amblyoma specific protein family 40-178 | 6 | 353 | 0.03 |
| Amblyoma specific protein family 40-177 | 5 | 459 | 0.04 |
| Amblyoma specific protein family 40-174 | 4 | 740 | 0.06 |
| Amblyoma specific protein family 40-172 | 4 | 3309 | 0.26 |
| Amblyoma specific protein family 40-166 | 3 | 135 | 0.01 |
| Amblyoma specific protein family 40-109 | 7 | 129 | 0.01 |
| Amblyoma specific protein abundantly expressed | 3 | 6197 | 0.49 |
| Amblyoma specific protein family 40-320 | 4 | 100 | 0.01 |
| Amblyoma specific protein family 35-455 | 3 | 50 | 0.00 |
| Amblyoma specific protein family 35-324 | 3 | 98 | 0.01 |
| Amblyoma specific protein family 35-314 | 4 | 552 | 0.04 |
| Amblyoma specific protein family 35-23 | 4 | 46 | 0.00 |
| Amblyoma specific protein family 35-220 | 2 | 306 | 0.02 |
| Amblyoma specific protein family 35-455 | 17 | 1686 | 0.13 |
| Amblyoma specific protein family 40-38 | 18 | 732 | 0.06 |
| Amblyoma specific protein family 35-90 | 7 | 2601 | 0.21 |
| Amblyoma specific protein family 35-506 | 3 | 38 | 0.00 |
| Amblyoma specific protein family 35-214 | 3 | 45 | 0.00 |
| Amblyoma specific protein family 35-20 | 8 | 709 | 0.06 |
| Amblyoma specific protein family 30-66 | 3 | 36 | 0.00 |
| Amblyoma specific protein family 30-209 | 3 | 145 | 0.01 |
| Amblyoma specific protein family 30-170 | 5 | 525 | 0.04 |
| Amblyoma specific protein family 30-123 | 4 | 72 | 0.01 |
| Amblyoma specific protein family 40-91 | 10 | 477 | 0.04 |
| Amblyoma specific protein family 45-15 | 18 | 772 | 0.06 |
| Amblyoma specific protein family 45-155 | 6 | 367 | 0.03 |
| Amblyoma specific protein family 45-555 | 3 | 215 | 0.02 |
| Amblyoma specific protein family 45-584 | 3 | 157 | 0.01 |
| Amblyoma specific protein family 45-1582 | 5 | 82 | 0.01 |
| Amblyoma specific protein family 40-56 | 11 | 1342 | 0.11 |
| Amblyoma specific protein family 40-72 | 7 | 281 | 0.02 |
| Amblyoma specific protein family 40-72 | 9 | 1579 | 0.13 |
| Amblyomma specific 9.2 kDa | 10 | 599 | 0.05 |
| Amblyomma specific 6.0 kDa | 5 | 91 | 0.01 |
| Other uncharacterized putative secreted proteins | 1118 | 41936 | 3.35 |
| **Unknown proteins or fragments** | 4167 | 160529 | 12.82 |
| **Transposable element products** |  |  |  |
| Class I | 142 | 2717 | 0.22 |
| Class II | 97 | 3241 | 0.26 |
| Other fragments | 77 | 1619 | 0.13 |
|  |  |  |  |
| **Total** | 15814 | 1251937 |  |
